# Clinical and Imaging Features of Chronic Occult Infectious Arthritis and Undifferentiated Oligoarthritis: A Comparative Analysis

**DOI:** 10.3390/jcm14176213

**Published:** 2025-09-03

**Authors:** Lingge Wu, Tao Chen, Yan Wang, Zhe Guo, Wangna Tang, Hong Zhao, Xueya Lv, Xiaoli Deng

**Affiliations:** 1 Department of Orthopedics Medicine, Beijing Jishuitan Hospital, Capital Medical University, 31 Xinjiekou East St, Xicheng District, Beijing 100035, China; wulingge@outlook.com (L.W.);; 2 Department of Ultrasound, Beijing Jishuitan Hospital, Capital Medical University, 31 Xinjiekou East St, Xicheng District, Beijing 100035, China; 3 Department of Laboratory Medicine, Beijing Jishuitan Hospital, Capital Medical University, 31 Xinjiekou East St, Xicheng District, Beijing 100035, China; 4 Department of Radiology, Beijing Jishuitan Hospital, Capital Medical University, 31 Xinjiekou East St, Xicheng District, Beijing 100035, China

**Keywords:** oligoarthritis, undifferentiated arthritis, infectious arthritis, ultrasound, serum albumin, imaging

## Abstract

**Background**: Undifferentiated arthritis is characterized by synovitis that does not meet the criteria for any specific rheumatic disease. However, a subset of chronic occult infectious arthritis, owing to atypical or overlapping clinical features, is often misclassified as undifferentiated oligoarthritis, potentially leading to diagnostic delays and suboptimal management. This study aimed to compare the clinical, laboratory, and imaging characteristics of these two types of oligoarthritis and to evaluate potential discriminatory markers. **Methods**: Patients older than 16 years with synovitis involving ≤2 joints at Beijing Jishuitan Hospital from September 2023 to December 2024 were included. Ultrasound-guided joint aspiration or synovial biopsy samples were analyzed by culture and next-generation sequencing, classifying patients as pathogen-positive or -negative. Clinical, laboratory, and imaging data (ultrasound, MRI, CT, X-ray) were compared, and multivariable logistic regression and ROC analyses were performed to identify predictors of infectious arthritis. **Results**: A total of 57 patients were included, with 20 (35.1%) categorized as pathogen-positive and 37 (64.9%) as pathogen-negative. The mean age was 41.7 ± 14.3 years, and 61.4% of the patients were female, with no significant demographic differences between groups. Monoarthritis was more common in pathogen-positive patients, accounting for 95% of cases (*p* = 0.02). Although the distribution of affected joints was similar between groups, ultrasound revealed a significantly higher bone erosion grade in pathogen-positive patients (*p* = 0.02), and CT/X-ray demonstrated articular surface destruction in 58.8% of infectious cases compared to 6.2% in pathogen-negative cases (*p* < 0.001). Serum albumin levels were significantly lower in the pathogen-negative group (20.7 ± 8.5 g/L vs. 41.1 ± 3.9 g/L, *p* < 0.001). ROC analysis determined that an albumin threshold >35.4 g/L predicted microbiological positivity with 100% sensitivity and 69.7% specificity. Multivariable logistic regression identified normal serum albumin levels, severe ultrasound-detected bone erosion, and imaging evidence of joint surface destruction as significant predictors of chronic occult infectious arthritis. **Conclusions**: Our findings suggest that, despite overlapping clinical and laboratory features, serum albumin levels, severe bone erosion on ultrasound and articular surface destruction on CT/X-ray may help differentiate chronic occult infectious arthritis from undifferentiated oligoarthritis. Further studies with larger cohorts are needed to confirm these preliminary results.

## 1. Introduction

Undifferentiated oligoarthritis, an early inflammatory arthritis that does not yet meet the diagnostic criteria for any specific arthritis, is characterized by inflammation affecting fewer than five joints. It accounts for approximately 30% of early arthritis cases [1]. A substantial proportion of patients with undifferentiated arthritis (UA) progress during follow-up to defined rheumatic diseases such as rheumatoid arthritis (RA), spondyloarthropathies, or systemic autoimmune disorders. In contrast, another subset of patients remain in a persistent UA state for years or eventually achieve spontaneous remission [2]. The pathophysiology of undifferentiated oligoarthritis is heterogeneous, primarily driven by immune-mediated mechanisms. Synovial inflammation results from the infiltration of immune cells, including T cells, B cells, and macrophages, which release pro-inflammatory cytokines such as tumor necrosis factor-alpha (TNF-α), interleukin-6 (IL-6), and interleukin-1β (IL-1β). These cytokines promote synovial hyperplasia, cartilage degradation, and bone erosion, contributing to joint damage [3]. The lack of specific autoantibodies or diagnostic markers in undifferentiated oligoarthritis complicates its classification, and it may represent an early stage of autoimmune diseases or a distinct entity with overlapping features [4].

However, chronic occult infectious arthritis is caused by low-virulence organisms such as Staphylococcus epidermidis, Propionibacterium acnes, or atypical mycobacteria, which evade host immune responses and establish persistent infections within the joint. The pathophysiology of infectious arthritis involves direct microbial invasion of the synovial membrane, triggering an inflammatory cascade. Pathogens stimulate the release of pro-inflammatory cytokines and matrix metalloproteinases (MMPs) from synovial cells, leading to cartilage degradation and bone erosion [5]. Low-virulence organisms often form biofilms, which are complex microbial communities encased in an extracellular matrix that enhances resistance to antibiotics and immune clearance [6]. Biofilm formation contributes to the chronicity of infection, as it shields pathogens from phagocytosis and promotes a smoldering inflammatory response without systemic signs like fever or leukocytosis [7,8,9]. It is possible for this to be misclassified as undifferentiated oligoarthritis

Undifferentiated arthritis presents with elevated inflammatory markers, but the absence of specific clinical features and reliable biomarkers prevents classification into a defined arthritis subtype [10]. Studies have shown that certain cases of infectious arthritis also exhibit elevated CRP levels, making differentiation from non-infectious arthritis challenging [11]. This overlap in clinical and laboratory profiles can lead to delayed diagnosis or inappropriate management, risking joint destruction in infectious cases or unnecessary antimicrobial therapy in non-infectious ones.

Although the critical need to accurately estimate the potential presence of chronic occult infectious in patients diagnosed with undifferentiated arthritis remains necessary, current research remains scarce. In this study, we analyze a cohort of patients with oligoarthritis and categorize them based on microbiological findings to further explore the clinical and imaging characteristics of patients with or without pathogens.

## 2. Materials and Methods

### 2.1. Patients

**Figure 1:**
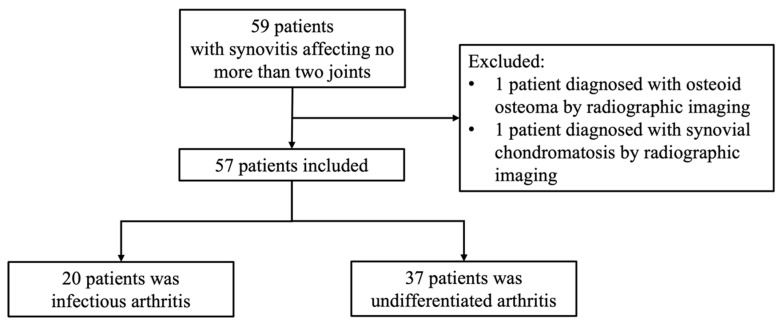
Flowchart of patient screening.

This study was designed as a cross-sectional analysis. Data of patients older than 16 years old who presented with synovitis affecting no more than two joints at the Department of Orthopedics Medicine, Beijing Jishuitan Hospital, from 1 September 2023, to 30 December 2024 were reviewed. To ensure greater comparability with infectious arthritis and reflect the characteristics of our cohort, we adopted this more restrictive definition (two or fewer joints). The inclusion criteria were as follows: (1) no history of relevant joint surgery; (2) absence of clinical features indicative of purulent arthritis, including continuous fever, acute onset (<3 months), or evidence of abscess or sinus tract formation; (3) did not meet the diagnostic criteria for any specific type of arthritis, including but not limited to rheumatoid arthritis, seronegative spondyloarthropathy, osteoarthritis, or crystal-induced arthritis (e.g., gout, calcium pyrophosphate dihydrate crystal deposition disease) by laboratory and imaging examinations; (4) underwent ultrasound-guided joint aspiration or synovial biopsy, with samples analyzed by microbiological cultures and next-generation sequencing (NGS) for pathogen detection; (5) completed imaging assessments, including ultrasound, X-ray, CT, or MRI. Patients with other pathological conditions contributing to synovitis, as determined by imaging and pathological findings, were excluded (Figure 1). Based on aspiration or biopsy results, patients with negative results for both pathogen tests were classified into a “pathogen-negative group”, while patients with a positive result for either of the pathogen testing methods were assigned to the “pathogen-positive group” following consensus discussions between clinical and microbiology specialists.

The study was conducted in accordance with the Declaration of Helsinki (as revised in 2013) and approved by the Ethics Committees of Beijing Jishuitan Hospital (No. K2024-281; date: 28 June 2024). Given that this study involves a review of patient records, written informed consent was waived.

### 2.2. Clinical Data

Patients’ demographic data include sex, age, body mass index (BMI), disease duration, history of infection, fever, and morning stiffness during the course of disease. The Visual Analog Scale (VAS) was utilized to assess and describe the patients’ joint pain. The reference ranges for all blood laboratory tests, including routine blood tests, ESR, CRP, immune globulin, albumin (Alb), bone metabolism markers, and antinuclear antibody (ANA) are provided in Supplementary Table S1. Joint examination was described based on the swelling, tender, warmth, and range of motion (ROM) limitation of the most severely affected joint.

All imagological examinations were conducted in the department of ultrasound and radiology in Beijing Jishuitan Hospital. The Doppler blood flow grading of ultrasound was categorized into four levels (0–3) according to the semi-quantitative scoring system by Terslev et al. [12]. Bone erosion levels were also classified into four stages (0–3) based on the report by Szkudlarek et al. [13]. Bone marrow edema and bone erosion semi-quantitative grading in magnetic resonance imaging (MRI) were categorized into four levels (0–3) and eleven levels (0–10), respectively, according to the rheumatoid arthritis-MRI Scoring System (RAMRIS) [14]. Notably, to accommodate cases where the affected joints were located at the distal ends of long bones, we defined the sensitive areas as the metaphysis of the long bones. For the sacroiliac joints, the sensitive area was defined based on the location of bone marrow edema or erosion, as either the iliac or sacral auricular surface. The ankle joint grading followed the same criteria as RAMRIS. The interpretation of ultrasound and imaging results and scores (including ultrasound, X-ray, computerized tomography [CT], and MRI) was performed under the supervision of experts in the respective fields.

### 2.3. NGS Workflow and Quality Control

Metagenomic next-generation sequencing (mNGS) was performed according to standardized protocols for low-biomass clinical samples. All procedures were conducted in a biosafety cabinet under a unidirectional workflow to minimize contamination, with strict physical separation of pre- and post-PCR areas. Synovial fluid samples underwent nucleic acid extraction using a magnetic bead-based method, followed by quantification with Qubit fluorometry. Library preparation included enzymatic fragmentation, end repair, adapter ligation, and PCR amplification. Libraries meeting quality control thresholds (≥0.5 ng/μL and acceptable fragment size distribution) were subjected to sequencing on the MGISEQ-200 platform.

Negative controls (sterile water and extraction blanks) and positive controls (spiked reference standards) were included in each batch to monitor assay performance and detect potential contamination. Sequence reads were quality-filtered, human host reads removed, and the remaining reads aligned to curated microbial reference databases. A pathogen was considered positive only if (i) its read number exceeded established thresholds validated by the laboratory, (ii) it was absent from negative controls, and (iii) it showed species-level alignment specificity. To ensure reproducibility, duplicate control samples were routinely processed, and all operators underwent standardized training and competency evaluation.

### 2.4. Statistical Analysis

Continuous variables were analyzed using *t*-tests or non-parametric tests, with *t*-test results expressed as mean ± standard deviation (SD) and non-parametric test results presented as median and interquartile range (IQR). Frequencies and percentages were reported for categorical variables and Pearson’s chi-squared and Fisher’s exact test were used for comparison between groups. The association between baseline characteristics and pathogen positivity status was assessed using multivariate logistic regression analysis that included all factors with *p* < 0.05 from a univariate analysis. The results of the multifactorial logistic regression were presented as the odds ratio (OR) and 95% confidence interval (CI). Receiver operating characteristic (ROC) curve analysis was performed to assess the diagnostic accuracy of predictive biomarkers and to determine the optimal cutoff values for distinguishing between infectious arthritis and undifferentiated oligoarthritis. The area under the ROC curve (AUC) was recorded, along with sensitivity and specificity at the optimal threshold. All statistical tests were 2-tailed, with the 2-sided statistically significant level set at *p* < 0.05. The statistical software was R 4.4.2.

## 3. Results

### 3.1. Baseline Characteristics Between Pathogen-Positive and Pathogen-Negative Group

A total of 57 patients with oligoarthritis who had microbiological results were included in the study, with 37 patients being microbiologically negative and 20 patients being microbiologically positive. The age ranged from 16 to 71 years, with a mean age of 41.7 ± 14.3 years, and 61.4% of the patients were female. Demographic characteristics, including gender, age, and BMI, showed no significant differences between the two groups. Seven patients (12.3%) had symptoms for less than 3 months before seeking medical attention, with 71.4% of these cases confirming the presence of a pathogenic microorganism. However, there was no significant difference in disease duration between the pathogen negative and positive groups (12 [IQR 6–36] months vs. 17 [IQR 3–30] months, *p* = 0.49). In terms of affected joints, 44 patients (22.8%) had involvement of one joint, and the proportion of monoarthritis was significantly higher in the pathogen-positive group compared to the pathogen-negative group (95% vs. 67.6%, *p* = 0.02). The most commonly involved joint in microbiologically negative patients with undifferentiated oligoarthritis was the knee joint (43.2%), followed by the wrist joint (40.5%). In patients with chronic occult infections, the most commonly affected joint was the ankle (30%), followed by the wrist and knee joints, each at 25%. However, no significant differences were observed between the two groups (*p* = 0.63). Both clinical symptoms (including fever, history of infection, diarrhea or tooth extraction, morning stiffness, and VAS) and those found via joint examination, like swelling, tenderness, ROM, and warmth, showed no significant differences between the two groups. Laboratory test results revealed that patients who tested positive for the pathogen had normal Alb levels (41.1 ± 3.9 g/L), whereas those oligoarthritis without microorganisms had significantly lower levels (20.7 ± 8.5 g/L, *p* < 0.001). No significant differences were observed in other inflammatory markers, bone metabolism indicators, or antinuclear ANA levels (Table 1).

Among the microbiologically positive patients, Staphylococcal infections were the most common, occurring in seven cases, with *Staphylococcus aureus* being the most frequent, accounting for three cases. The most commonly identified microorganism was *Mycobacterium tuberculosis*, which was detected in four cases. Details of other microbial species are provided in Table 2.

### 3.2. Imaging Features in Pathogen-Negative and -Positive Patients

Imaging assessments of microbiologically negative and positive patients were primarily conducted using ultrasound, MRI, CT, or X-ray. Ultrasound evaluation focused on synovial thickness, Doppler blood flow signals, bone erosion extent, and the presence of joint effusion. No significant differences were observed between the two groups in terms of synovial thickness, blood flow signal intensity, or effusion in the most severely affected joint. However, the bone erosion grade was significantly higher in microbiologically positive patients compared to pathogen-negative patients (*p* = 0.02). Bone erosion was detected in 75% of the affected joints of microbiologically positive patients on ultrasound, while only 32.4% of microbiologically negative patients exhibited bone erosion. Although the proportion of patients with bone marrow edema in MRI was higher in the pathogen-positive group compared to the negative group (90% vs. 69.6%), and bone erosion was also more frequent (60% vs. 47.8%), these differences did not reach statistical significance. CT or X-ray revealed joint surface damage in 58.8% of patients with the pathogen, a significantly higher proportion compared to 6.2% in oligoarthritis patients without the microorganism (*p* < 0.001). Similarly, joint space narrowing was observed in 65% of participants within the pathogen-positive group, compared to 35.3% of patients without the pathogen, although this difference did not reach statistical significance (*p* = 0.07).

### 3.3. Factors for Positive Pathogen

A multivariable logistic regression analysis was conducted to identify potential factors associated with pathogen-positive arthritis. Serum Alb levels, ultrasound bone erosion grade, and joint surface damage observed on CT or X-ray were included in the analysis. The results indicated that for every 1 g/L increase in serum Alb, the likelihood of being microbiologically positive decreased by 0.64 times (95% CI 0.45–0.90, *p* = 0.002). We further assessed the relationship between serum albumin and other inflammatory markers or disease duration using Spearman’s correlation analysis. No significant correlations were observed in albumin with other inflammatory markers or disease duration.

Compared to patients without joint surface damage, those with joint surface destruction had an increased possibility of being microbiologically positive (OR 1.61, 95% CI 1.20–2.15, *p* = 0.003). Among ultrasound reports of bone erosion, patients with grade 3 bone erosion had a significantly higher risk of being microbiologically positive compared to those with smooth bone surfaces (OR 1.38, 95% CI 1.02–1.87, *p* = 0.045), while grade 1 and 2 bone erosion did not provide a reliable indication of microbiological positivity (Table 3).

**Figure 2:**
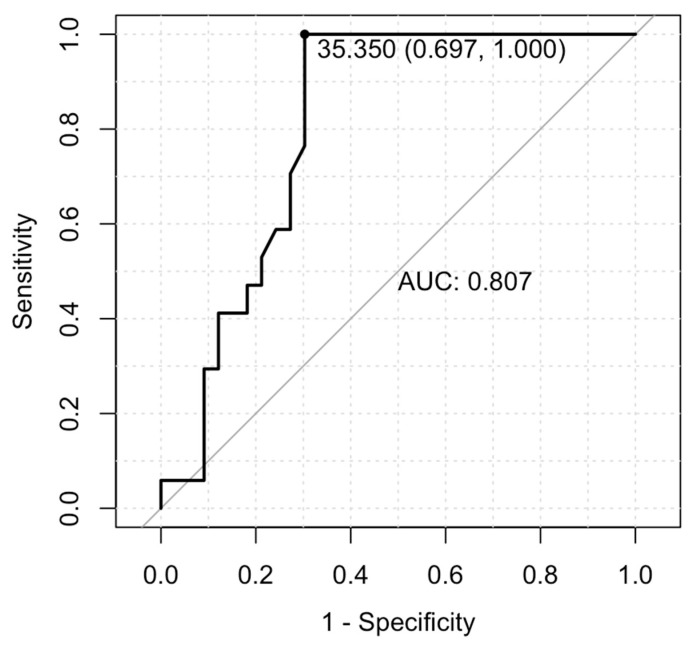
ROC curve for serum albumin as a predictor of pathogenic infection in oligoarthritis. ROC—receiver operating characteristic; AUC—area under curve.

ROC curve analysis was used to determine the threshold value of Alb for predicting the presence of pathogenic microorganisms in patients with oligoarthritis. It was found that when Alb levels exceeded 35.4 g/L, the specificity for predicting microbiological specificity in oligoarthritis patients was 69.7%, with a sensitivity of 100% (Figure 2).

## 4. Discussion

This comparative study analyzed the clinical characteristics of patients with undifferentiated oligoarthritis, comparing those with and without detectable pathogens. In clinical practice, some patients with chronic occult infections are misclassified as having undifferentiated arthritis due to their atypical presentation, leading to treatment with disease-modifying antirheumatic drugs (DMARDs) and other immunosuppressive therapies, often with suboptimal efficacy. The diagnosis of chronic occult joint infections typically requires invasive procedures such as joint aspiration or synovial biopsy for microbiological testing, contributing to increased healthcare costs. Therefore, identifying clinical and imaging characteristics that aid in distinguishing infectious from non-infectious arthritis is of critical importance. Our findings suggest that decreased serum Alb levels may serve as a predictive factor for undifferentiated oligoarthritis without a pathogen, whereas demographic characteristics, inflammatory markers, and antinuclear antibody positivity showed no significant differences between the two groups. In terms of imaging features, ultrasound findings of more severe bone erosion and CT/X-ray evidence of articular surface destruction were more suggestive of pathogen-positive arthritis.

Differentiating infectious arthritis from undifferentiated arthritis based on medical history, clinical presentation, and physical examination is often challenging. Gupta et al. conducted a study in adult oligoarthritis patients in India and found that although no specific clinical features distinguished infectious arthritis from inflammatory arthropathies such as gout, systemic lupus erythematosus, and early rheumatoid arthritis, the prevalence of erosive arthritis was significantly higher in the infectious arthritis group than in the non-infectious arthritis group (*p* < 0.05). Moreover, ESR and CRP levels were also significantly elevated in the infectious arthritis group compared to the non-infectious arthritis group [15]. In our study, we found that more severe bone erosion on ultrasound was more indicative of joint infection, which is similar to the findings presented in previous research. However, we did not observe abnormal ESR or CRP levels in our cohort, which may be attributed to the relatively low virulence of pathogens in our patient population.

Regarding serological markers, ANA showed extremely poor discriminative ability between the two groups (*p* > 0.99), and all ANA-positive cases in our cohort exhibited low titers (1:100). This may be related to the fact that low-titer ANA positivity can also be detected in the healthy population in China [16]. It may also represent subclinical autoimmunity or transient reactivity and should therefore be interpreted with caution. No significant differences in immunoglobulin levels were observed between the two groups. Although hypoalbuminemia is generally suggestive of systemic inflammation or infection, in our study, pathogen-positive patients exhibited albumin values (~41 g/L) within or close to the normal range, whereas pathogen-negative patients had markedly reduced levels (~21 g/L). Importantly, no correlation was found between serum Alb levels and other parameters such as ESR, CRP, or disease duration, suggesting that albumin may provide independent discriminative value in this context. To date, no studies have reported findings similar to ours. A previous study indicate that albumin levels are reduced during acute arthritis, a phenomenon attributable to the suppression of hepatic synthesis by inflammatory cytokines and to increased catabolic clearance [17]. However, in rheumatoid arthritis, reduced Alb synthesis and increased degradation due to chronic inflammation resulted in lower serum levels [18]. In our cohort, patients with culture-negative disease likely represent an early phase of inflammatory arthritis, characterized by a pronounced systemic inflammatory response. In contrast, patients with low-virulence infections do not exhibit the overt inflammatory state typically seen in septic arthritis—evidenced by their relatively low systemic inflammatory marker levels. Based on these findings, we speculate that even in the infection group where local chronic inflammation is present, the systemic inflammatory burden associated with low-virulence infections appears to be ‘milder’ and less sustained than in undifferentiated oligoarthritis, and therefore may not suppress albumin synthesis and homeostasis as broadly or persistently. Moreover, serum albumin levels may also be influenced by nutritional status or metabolic conditions, which could confound their role as infection-specific markers. Overall, Alb may reflect the inflammatory status of the disease, but its utility in distinguishing chronic occult infectious arthritis from undifferentiated oligoarthritis requires longitudinal data and future work to assess the stability of albumin across the disease course and treatment response. A previous study on the MRI characteristics of infectious arthritis and juvenile idiopathic arthritis in pediatric patients found that bone marrow edema was more common in infectious patients (92% vs. 52%), which is consistent with our findings (90% vs. 69.6%) [19]. However, our study did not demonstrate a statistically significant difference, possibly due to the limited sample size. In addition to bone marrow edema, bone erosion has also been recognized as a characteristic feature of patients with a pathogen. Although MRI findings in our study indicated a higher proportion of bone erosion in the pathogen-positive group, the difference was not statistically significant, which may be attributed to the low-virulence nature of the pathogens in our cohort. Interestingly, bone erosion in ultrasonography demonstrated superior discriminatory ability in identifying pathogen-positive cases, particularly in severe bone erosion. As a convenient and widely used imaging examination, ultrasound plays an important role in clinical practice. Gray-scale ultrasound for assessing synovial thickening and power Doppler for evaluating synovial vascularity have been applied with increasing frequency in the early diagnosis of rheumatoid arthritis and the assessment of infectious arthritis [20,21]. Although severe bone erosion and articular surface destruction were more prevalent in pathogen-positive patients in our study, these findings are not specific to infection, as similar changes may occur in osteoarthritis and long-standing inflammatory arthritis. Therefore, imaging results should be interpreted in conjunction with microbiological and clinical findings. Additionally, despite standardized scoring, inter-operator variability and reproducibility remain potential limitations of semi-quantitative imaging assessment. Technical factors such as ultrasound resolution, joint morphology, and operator expertise may affect sensitivity in detecting erosions, and should be considered when interpreting these results. But to date, no studies have specifically investigated the use of ultrasound in singling out pathogen-positive patients from those with undifferentiated oligoarthritis. Larger multicenter studies are needed to validate our proposed albumin cut-offs and the predictive value of ultrasound-detected erosions. Further research is warranted to explore its potential role in distinguishing low-virulence microorganisms from undifferentiated oligoarthritis.

In our cohort, all patients underwent echocardiographic evaluation during hospitalization, and no evidence of infective endocarditis was detected. Five patients with microbiological results developed fever, and there were no records of positive blood cultures. These findings suggest that while septic arthritis can arise from bacteremia similar to bacterial endocarditis, such dissemination was not evident in our series. Given that these infections often lack classical clinical features of septic arthritis, we included them in our analysis. Low-virulence germs are typically characterized by strong biofilm-forming capacity and high immune evasion potential, often leading to chronic or insidious infections [22]. Biofilm formation may render bacteria more resistant to clearance, prolonging infection and facilitating chronic adaptation, and allowing them to persist in the joint environment with minimal systemic signs. *Staphylococcus aureus*, a known biofilm-forming pathogen, shares this characteristic [23]. These pathogens are more frequently associated with infections in immunocompromised individuals or patients with joint implants [24], whereas reports of native joint infections (NJIs) caused by low-virulence pathogens are relatively rare. A study comparing periprosthetic joint infection (PJI) and NJI found that *Staphylococcus* spp. were the most common causative pathogens in both types of infectious arthritis [25], which is consistent with our results. Compared with prosthetic joint infections, where biofilm biology is well recognized, biofilm-driven pathology in native joint infections may share similar mechanisms. Additionally, our study revealed a relatively high proportion of tuberculosis-associated arthritis, likely reflecting the higher prevalence of tuberculosis in China than in many Western countries [26]. The relatively high proportion of tuberculosis-related arthritis observed in our cohort may therefore reflect regional epidemiological patterns. All patients in our cohort had no severe underlying diseases leading to immunosuppression, and the source of infection in NJI cases remains unclear. A previous study on anaerobic bacterial arthritis suggested that such infections might be linked to dental procedures, such as tooth extractions or periodontitis [27]. However, none of the patients with anaerobic infections in our study reported any history of dental diseases or extraction. The routes of infection require further investigation. Overall, distinguishing pathogen positive and negative patients in undifferentiated oligoarthritis remains challenging due to their overlapping clinical presentations, despite their markedly different treatment strategies. Delayed or inadequate treatment may result in irreversible joint dysfunction and significantly impair quality of life. Therefore, a comprehensive diagnostic approach integrating synovial fluid analysis, microbiological cultures, and molecular detection techniques is essential to improve diagnostic accuracy and guide appropriate management.

This study has several limitations. First, the sample size was relatively small, which may have affected the stability of multivariate regression and ROC curve analyses. The absence of longitudinal follow-up data, including treatment response and outcomes, limits the clinical applicability of our findings. Second, complete imaging data were not available for all patients, as some lacked MRI, CT, or X-ray results, potentially affecting the comprehensiveness of the imaging analysis. Third, although each arthritis subgroup included various joints that are all synovial and are known to exhibit similar inflammatory responses, anatomical differences among these joints may have influenced the imaging characteristics and inflammatory patterns.

## 5. Conclusions

Differentiating chronic occult infectious arthritis from undifferentiated oligoarthritis remains challenging and clinicians should integrate multiple diagnostic modalities for comprehensive evaluation. Aspiration of the joint, microbiological culture, and complementary methods such as high-throughput sequencing and serological testing remain the mainstays of diagnosis, while the findings of this study highlight that atypical presentations may require aspiration and reconsideration of the diagnosis. Severe bone erosion in ultrasound and imaging evidence of articular surface destruction are associated with pathogen-positive oligoarthritis. Serum Alb may serve as a potential discriminatory marker.

## Figures and Tables

**Table 1:** Baseline characteristics of oligoarthritic patients with or without pathogen.

	Pathogen Negative (*n* = 37)	Pathogen Positive (*n* = 20)	*p* Value
Female (*n*, %)	24 (64.9)	11 (55.0)	0.66
Age (years, median [IQR])	39 [30, 47]	46 [35, 59]	0.23
BMI (kg/m^2^, median [IQR])	23.5 [21.8, 26.8]	22.3 [20.8, 25.4]	0.23
Course of disease (months, median [IQR])	12 [6, 36]	17 [3, 30]	0.49
VAS (median [IQR])	6 [5, 8]	6 [5, 8]	0.65
History of infection, diarrhea or stomatology operations (*n*, %)	5 (17.2)	6 (37.5)	0.25
History of fever (*n*, %)	6 (18.2)	5 (26.3)	0.74
Number of affected joints			
One joint	25 (67.6)	19 (95.0)	0.02
Two joints	12 (32.4)	1 (5.0)	
Morning stiffness (*n*, %)	8 (26.7)	7 (43.8)	0.40
Location of Affected Joints			
Wrist joint (*n*, %)	15 (40.5)	5 (25.0)	0.63
Elbow joint (*n*, %)	4 (10.8)	1 (5.0)	
Shoulder joint (*n*, %)	0 (0)	1 (5.0)	
Knee joint (*n*, %)	16 (43.2)	5 (25.0)	
Hip joint (*n*, %)	2 (5.4)	1 (5.0)	
Ankle joint (*n*, %)	2 (5.4)	6 (30.0)	
Sacroiliac joint (*n*, %)	0 (0)	2 (10.0)	
Joint examination			
Swelling (*n*, %)	32 (86.5)	18 (94.7)	0.63
Tenderness (*n*, %)	27 (75.0)	19 (95.0)	0.13
Decreased ROM (*n*, %)	30 (81.1)	20 (100.0)	0.10
Warmth (*n*, %)	20 (60.6)	13 (72.2)	0.60
ESR (mm/h, mean ± SD)	25.14 ± 27.36	40.1 ± 35.1	0.09
CRP (mg/L, mean ± SD)	10.67 ± 20.40	21.3 ± 37.3	0.18
Routine bloods			
White blood cell (×10^9^/L, mean ± SD)	6.4 ± 2.3	6.4 ± 1.7	0.98
Neutrophil (×10^9^/L, mean ± SD)	5.9 ± 11.0	3.8 ± 1.1	0.42
Lymphocyte (×10^9^/L, mean ± SD)	1.94 ± 0.72	1.96 ± 0.66	0.94
Monocyte (×10^9^/L, mean ± SD)	2.6 ± 11.0	0.4 ± 0.2	0.39
Platelet (×10^9^/L, mean ± SD)	270.5 ± 78.5	290.3 ± 94.6	0.42
Globulin			
Immune globulin G (g/L, mean ± SD)	14.0 ± 3.9	12.2 ± 3.2	0.15
Immune globulin A (g/L, mean ± SD)	2.8 ± 1.4	2.6 ± 1.2	0.73
Immune globulin M (g/L, mean ± SD)	1.3 ± 1.0	1.0 ± 0.6	0.39
Serum calcium (mmol/L, mean ± SD)	2.3 ± 0.1	2.3 ± 0.1	0.85
Serum phosphate (mmol/L, mean ± SD)	1.1 ± 0.2	1.2 ± 0.2	0.77
Alb (g/L, mean ± SD)	20.7 ± 8.5	41.1 ± 3.9	<0.001
Alkaline phosphatase (U/L, mean ± SD)	86.7 ± 35.0	92.6 ± 30.7	0.54
Total procollagen type 1 N-terminal propeptide (ng/mL, mean ± SD)	57.7 ± 32.3	62.9 ± 25.7	0.61
β-C-terminal telopeptide of type-I collagen (ng/mL, mean ± SD)	0.5 ± 0.3	0.7 ± 0.5	0.16
Parathyroid hormone (pg/mL, mean ± SD)	31.8 ± 13.4	31.3 ± 13.9	0.91
25-hydroxyvitamin D_3_ (ng/mL, mean ± SD)	19.8 ± 6.5	19.1 ± 7.1	0.71
ANA positive (n, %)	13 (35.1)	6 (33.3)	>0.99
Ultrasound			
Synovial thickness (cm, mean ± SD)	0.6 ± 0.5	0.8 ± 0.7	0.18
Doppler blood flow			
Level 0 (*n*, %)	8 (21.6)	9 (52.9)	0.12
Level 1 (*n*, %)	8 (21.6)	3 (17.6)	
Level 2 (*n*, %)	16 (43.2)	3 (17.6)	
Level 3 (*n*, %)	1 (2.7)	2 (11.8)	
Bone erosion			
Level 0 (*n*, %)	20 (54.1)	2 (11.8)	0.02
Level 1 (*n*, %)	7 (18.9)	9 (52.9)	
Level 2 (*n*, %)	2 (6.2)	1 (5.9)	
Level 3 (*n*, %)	3 (8.1)	5 (29.4)	
Effusion (*n*, %)	29 (78.4)	14 (70.0)	0.71
MRI			
Bone marrow edema			
0 (*n*, %)	7 (18.9)	1 (5.0)	0.36
1 (*n*, %)	3 (8.1)	2 (11.8)	
2 (*n*, %)	6 (16.2)	3 (17.6)	
3 (*n*, %)	7 (18.9)	4 (10.8)	
Bone erosion			
0 (*n*, %)	12 (32.4)	4 (20.0)	0.25
1 (*n*, %)	7 (18.9)	3 (17.6)	
2 (*n*, %)	3 (8.1)	1 (5.9)	
3 (*n*, %)	0 (0)	2 (11.8)	
4 (*n*, %)	1 (2.7)	0 (0)	
CT or X ray			
Joint space narrowing (*n*, %)	12 (35.3)	9 (65.0)	0.07
Joint surface destruction (*n*, %)	2 (6.2)	10 (58.8)	<0.001

BMI—body mass index; IQR interquartile range; VAS—Visual Analog Scale; ROM—range of motion; ESR—erythrocyte sedimentation rate; CRP—C-reactive protein; SD—standard deviation; Alb—albumin; ANA—antinuclear antibody; CT—computerized tomography; MRI—magnetic resonance imaging.

**Table 2:** Pathogen types in patients with positive microbiological findings.

Pathogen Spectrum	Count
Mycobacteria	
*Mycobacterium tuberculosis*	4
Gram-positive bacteria	
*Staphylococcus aureus*	3
*Staphylococcus capitis*	2
*Staphylococcus cohnii*	1
*Staphylococcus epidermidis*	1
*Corynebacterium jeikeium*	1
*Peptostreptococcus anaerobius*	1
Gram-negative bacteria	
*Brucella* spp.	2
*Escherichia coli*	1
*Fusobacterium nucleatum*	1
*Pseudomonas aeruginosa*	1
*Xylophilus ampelinus*	1
Virus	
Hepatitis B Virus	1

**Table 3:** Association between biochemical and imaging factors and pathogen positivity in oligoarthritis.

Facror	Pathogen-Positive OR (95%CI)	*p* Value
Alb (g/L)	0.64 (0.45, 0.90)	0.002
Bone erosion in ultrasound		
Level 1	1.22 (0.93, 1.59)	0.16
Level 2	0.95 (0.55, 1.62)	0.84
Level 3	1.38 (1.02, 1.87)	0.045
Joint surface destruction in CT or X ray	1.61 (1.20, 2.15)	0.003

OR—odds ratio; CI—confidence interval; Alb—albumin.

## Data Availability

The raw data supporting the conclusions of this article will be made available by the authors on request.

## Supplementary Materials

The following supporting information can be downloaded at: https://www.mdpi.com/article/10.3390/jcm14176213/s1, Table S1: Reference Ranges for Serum Laboratory Tests.
