# Supplementary material for: Clinical and Imaging Features of Chronic Occult Infectious Arthritis and Undifferentiated Oligoarthritis: A Comparative Analysis
Source: J Clin Med. 2025 Sep 3;14(17):6213. doi: 10.3390/jcm14176213 (PMC12429118; doi:10.3390/jcm14176213)
Supplement: Supplementary file 1 [file jcm-14-06213-s001.zip › jcm-3829569-SI.pdf]

Supplement

Table S1 Reference Ranges for Serum Laboratory Tests

Laboratory Test	Reference Range
ESR	0-20mm/h
CRP	0-8mg/L
White blood cell	3.5-9.5×10^9/L
Neutrophil	1.8-6.3×10^9/L
Lymphocyte	1.1-3.2×10^9/L
Monocyte	0.1-0.6×10^9/L
Platelet	120-350×10^9/L
Immune globulin G	7.51-15.6g/L
Immune globulin A	0.82-4.53g/L
Immune globulin M	0.46-3.04g/L
Serum calcium	2.11-2.52mmol/L
Serum phosphate	0.85-1.51mmol/L
Alb	40.0-50.0g/L
Alkaline phosphatase	50-135IU/L
Total procollagen type 1 N-terminal propeptide	13.1-58.5ng/ml
β-C-terminal telopeptide of type-I collagen	<1.008ng/ml
Parathyroid hormone	15-65pg/ml
25-hydroxyvitamin D_3	20-40ng/ml

ESR, erythrocyte sedimentation rate; CRP, C-reactive protein; Alb, albumin.
